# The Institutional Library

**Published:** 1919-02-01

**Authors:** 


### THE INSTITUTIONAL LIBRARY.

### THE CASUALTY CLEARING STATION.
### TRIUMPHS OF TRANSPORT AND SURGERY.

Major-General Cuthbert Wallace, C.M.G., F.R.C.S., and Captain John Fraser, M.C., F.R.C.S., have presented to the profession an extremely well written account* of the wonderful advances in the care and treatment of our wounded soldiers on the Western Front. The evolution of the Casualty Clearing Station is traced from the Field Hospital of the South African War, and from the Clearing Hospital of the period of reconstruction after it, to the modern Field Unit, the designation of which in the early days of the war, was changed to Clearing "Station," in order to obviate that misconception of its functions so liable to be associated with the term "Hospital." It must be borne in mind, however, that this volume was written during, and is applicable only to, the period of trench warfare; during the more recent period of open warfare the C.C.S. resumed its mobile character and its purely clearing functions. Incidentally these functions would be more appropriately expressed by the title "Casualty Clearing Ambulance," an item which will doubtless receive consideration during the period of reconstruction. The general policy of the Army Medical Authorities disclosed throughout the volume is one of unremitting endeavour, by research and by the devoted labours and self-sacrifice of scientists, doctors and nurses, to ensure for our wounded the most up-to-date treatment at the earliest possible moment after the infliction of the wounds. The goal aimed at is ideal: prevention, commencing on the battlefield, is the keynote—the prevention of pain, shock, sepis, deformity and of complications, such as tetanus, gas gangrene, and prolonged suppuration, many of the new methods being revolutionary.

The organisation in advance of the Clearing Station is charged with the duty of collecting the wounded and delivering them in the best possible condition for operation at the earliest possible moment. At the extreme front, limbs are fixed and extended in Thomas' splints, and suspended from bars which are attachable to the stretchers; body heat is maintained by blankets and by hot-water bottles or hot bricks, by hot drinks en route, at the regimental aid posts, and advanced dressing stations, and by the heating of ambulance transport. The rapid transport which is so essential is ensured by sending up wheeled stretchers, trench railway ambulance trains, and motor ambulance wagons, to relieve hand carriage as near to the front as the roads will permit. This organisation has met with marked success, for in comparison with the earlier days of the war there has been a decrease in mortality from shock, of those arriving at clearing stations, of 25 per cent.

The Clearing Station of to-day is very different from its original model. Its nominal capacity is 1,000 beds, 800 of which are on a "clearing" basis, with stretcher beds, and 200 on a "hospital" basis, with beds, nurses, special departments, such as X-ray, bacteriological, and resuscitation, and operation rooms with teams, often ten to twelve in number, each consisting of two surgeons, an anæsthetist, a nursing sister, and trained R.A.M.C. operation-room attendant.

* *Surgery at a Casualty Clearing Station.* By Cuthbert Wallace, C.M.G., F.R.C.S. (London, A. C. Black, Ltd., 1918. Pp. 311. Price 10s. 6d.)

The type of surgical work which is associated with the Casualty Clearing Station is very lucidly summarised. There are separate chapters on surgical shock, antiseptics, transfusions, tetanus, gas gangrene, and the gunshot injuries of bones, joints, nerves, blood-vessels, eyes, and each region of the body.

Surgical shock is fully discussed. The theories of origin are as numerous as cures for consumption, and finality is as far off as ever; the most powerful predisposing causes are considered to be "nervous temperament" and "cold," and the possible sequence of events, a rapid imperceptible pulse, a low blood pressure, a depressed body temperature, and a decreased alkalinity of the blood and body tissues. In the treatment, warmth is the main stand-by, electrically-heated bed cradles being included in the equipment of the resuscitation wards. The majority of cases either recover or materially improve in the cradle, in an hour. If they do not do so, efforts are directed towards raising the blood pressure, the method most widely adopted being the transfusion of compatible human blood, or in the absence of donors, a six to seven per cent. solution of gum acacia. In the campaign against wound infection the older antiseptics, such as carbolic and mercury, have proved disappointing, and the idea that dirty, lacerated wounds could be sterilised by isolated applications of powerful antiseptics had early to be abandoned. Hypochlorous acid heads the list of the new war antiseptics; the various methods of its preparations are fully described. Excellent results have also been obtained with the di-chloramines, synthetic dyes, including "flavine," Wright's salt solutions, and pastes, such as Rutherford Morrison's B.I.P.P.

The chapters on gunshot injuries are full of interest, abounding as they do with novel ideas and changed principles. Approximately 50 per cent. of wounds admitted to a C.C.S. are now treated by excision, disinfection, and primary suture, either immediate or delayed (24-72 hours), good results being obtained in 70 per cent. Open comminated fractures, which in previous wars were treated by amputation, are now conservatively and successfully treated by excision of the wound, removal of foreign bodies and infected tissues, sterilisation, immobilisation, and primary or secondary closure, new and ingenious methods of splinting and extension contributing essentially to the success of the method. The advance in abdominal surgery is exemplified by statistics, the operative mortality having been reduced to 51.3 per cent. Bomb wounds result in the lowest mortality (49 per cent.), shell and bullet wounds come next (51 per cent.), and shrapnel cause 70 per cent. Time is a vital factor. The majority of the cases receive operative attention between six and ten hours after the receipt of the injury; up to six hours the chances are in favour of the patient; after this period they are always against him. The principle of treatment in specialised departments, of all regional injuries, has in no instance been more fully justified than in wounds of the head, the previous very great early mortality having been very materially reduced by progressive improvements in operative technique. The introduction of the steel helmet was followed by a relative reduction in the number of head wounds and of the proportion of penetrating as compared with non-penetrating wounds.

**Institutional Library** (*continued*).

Badly infected wounds were also considerably diminished. In the early months of the war it was found that the incidence of tetanus among the wounded was higher than had been anticipated. This was due to the highly-manured soil, in which tetanus spores abounded, the infection by which of every article of clothing was favoured by the conditions of trench warfare. In the British Army the prophylactic inoculation of antitetanic antitoxin was administered at the earliest possible moment after the receipt of the wound and repeated afterwards; in the French Army, antitetanic vaccination was practiced in addition, the details of which have already been published in THE HOSPITAL of December 7, 1918, page 195. Anæsthetics which have played so important a rôle in the work of the C.C.S. are not mentioned, the general use of gas and oxygen, for instance, has saved many a hopeless case and prevented mortality from shock.

The book, however, is an excellent one, and will be a great help to Army surgeons.

### ARTHROMETRY: THE MEASUREMENT OF THE MOBILITY OF JOINTS.

THAT for their services in the greatest of wars our glorious Navy, Army, and Air Service deserve well of their country is endorsed by a nation full of gratitude to the sound, and of sympathy for the disabled. The wonderful triumphs of surgery; the prevention of shock, by rapid transport, maintenance of body heat and improvements in anæsthetics; the prevention of sepsis, and the conservation of limbs, by changed methods of treatment such as excision of wounds, sterilisation, primary suture, and immobilisation by specialised splinting, have been fully described in THE HOSPITAL (The Great War Series—The Casualty Clearing Station). Equally important is the after-treatment of our wounded; this also is making rapid strides towards the ideal; hospitals for physical treatment are developing, and institutions for the supply and fitting of artificial limbs are in a forward state. In no department of restoration of the disabled has greater progress been made than in that of physical treatment: fresh ideas, new methods, and changed principles, born of experience and necessity, are constantly engaging the attention of the highest intellects of the profession.

It is opportune, therefore, that the means of recording progress should keep pace with treatment. We are indebted to Mr. W. Wilbraham Falconer* for a method of measuring and recording the progress of those under treatment for disabled limbs which is simple, rapid, effective, and inexpensive. The scientific measurement of the mobility of joints has not previously received general attention in England. The French have a system which has many advantages, but the various instruments in use both there and in this country are inaccurate, slow, difficult in use, and expensive. In the conception of his instrument Falconer has adapted the simple principle of gravity; the arthrometer consists of a circular plated box, 4 in. by 1 in., fitted with a central spindle, to one end of which is attached a pointer, which works on a revolving dial marked with 180°, and to the other end, within the box, a weighted arm. For attachment, there is a metal and leather strap, which can be moulded to conform to the shape of any limb. The indicator, by means of the weighted pendulum, retains a vertical position, and the dial assumes a position depending upon the nature of the movement imparted to the limb. Mr. Falconer's method is more than a simple system of mensuration, it is a unified system of testing and of recording the mobility of joints; it includes a list of correct positions for the observation of their movements, and a table showing the average range of mobility possessed by almost every joint in the body. An illustration is also given by one of a series of arthrometrical charts on which is diagramatically recorded the result of the gradual relaxation of a stiff knee joint, it is a most ingenious device on which the history of two months treatment is seen at a glance on the space of a few square inches. The little book, apart from the instructions for the use of the invaluable arthrometer, contains knowledge which is essential for every practitioner and every nurse. Mr. Falconer has placed at our disposal, as the result of his great experience at the Red Cross Clinic for Physical Treatment, a very valuable link in the chain of restoration of our wounded.

**Arthrometry, or The Measurement of the Movements of Joints.* By W. Wilbraham Falconer. Superintendent of mechanical treatment at the Red Cross Clinic for the physical treatment of disabled officers, Great Portland Street, London, W. (London: John Bale, Sons & Danielson, Ltd., 1918).

**Casting out Fear.** By FLORA BIGELOW GUEST. Second edition. (London: John Lane, The Bodley Head. 1918. Price 3s. 6d. net.)

WITHOUT wishing to be unkind, the present writer records his opinion that this is one of the silliest books which he has ever involuntarily read. But when, on page 11, it is asserted that "the fear of death poisons the joy of life," in face of the fact that the fear of living afterwards has not deterred the authoress from writing these gratuitous statements, what else can be said? It is a pitiful series of ineptitudes, to which hardly a moment's attention, a flash of insight, or a trace of humour, has contributed. It must have delighted some people, however, for we note that the present is described as the second edition. No doubt the cookery books which the authoress has written are excellent and practical: "Soup, Oysters, and Surprises" is in itself an invitation, and what delightful prospect is not opened by a work on "Fancy Breads"? Let us hope that the publication of the latter books was as judicious as that of the present work is ill-advised. A writer who warns us against the "fear of consequences" will excuse a candid, though invited, expression of opinion which is sincere but does not mean to be unfriendly.

**Clinical Case-Taking.** By ROBERT D. KEITH, M.A., M.D. (London: H. K. Lewis and Co. 1918. Pp. 104. Price 3s. 6d. net.)

THIS is not an ambitious book, but it is distinguished by very useful qualities. Its aim is to serve as a companion and guide to the student entering on a course of clinical medicine. It endeavours to teach a right method of examination and to describe the evidences of disease as these are met at the bedside. By following the directions supplied the novice will become habituated to a sound and systematic procedure, and will learn to observe and to interpret the signs and symptoms of disease. The book is very carefully written, and within the limits which the author has prescribed for himself he has achieved a decided success.

